# circMTND5 Participates in Renal Mitochondrial Injury and Fibrosis by Sponging MIR6812 in Lupus Nephritis

**DOI:** 10.1155/2022/2769487

**Published:** 2022-10-11

**Authors:** Junjun Luan, Congcong Jiao, Cong Ma, Yixiao Zhang, Xiangnan Hao, Guangyu Zhou, Jingqi Fu, Xingyu Qiu, Hongyu Li, Wei Yang, Gabor G. Illei, Jeffrey B. Kopp, Jingbo Pi, Hua Zhou

**Affiliations:** ^1^Department of Nephrology, Shengjing Hospital of China Medical University, Shenyang, China; ^2^Department of Urology, Shengjing Hospital of China Medical University, Shenyang, China; ^3^Program of Environmental Toxicology, School of Public Health, China Medical University, Shenyang, China; ^4^Department of Physiology, School of Basic Medical Sciences, Zhejiang University School of Medicine, Hangzhou, China; ^5^Shensu Science & Technology Co. Ltd., Suzhou, China; ^6^Horizon, Gaithersburg, MD, USA; ^7^Kidney Disease Section, NIDDK, NIH, Bethesda, USA

## Abstract

Recent studies have focused on nuclear-encoded circular RNAs (circRNAs) in kidney diseases, but little is known about mitochondrial circRNAs. Differentially expressed circRNAs were analyzed by RNA deep sequencing from lupus nephritis (LN) biopsies and normal human kidneys. In LN renal biopsies, the most downregulated circRNA was circMTND5, which is encoded in the mitochondrial genome. We quantitated circMTND5 by qPCR and localized by fluorescence in situ hybridization (FISH). Mitochondrial abnormalities were identified by electron microscopy. The expression of mitochondrial genes was decreased, and the expression of profibrotic genes was increased on qPCR and immunostaining. RNA binding sites for MIR6812 and circMTND5 were predicted. MIR6812 expression was increased by FISH and qPCR. In HK-2 cells and its mitochondrial fraction, the role of circMTND5 sponging MIR6812 was assessed by their colocalization in mitochondria on FISH, RNA immunoprecipitation, and RNA pulldown coupled with luciferase reporter assay. circMTND5 knockdown upregulated MIR6812, decreased mitochondrial functional gene expression, and increased profibrotic gene expression. Overexpression of circMTND5 reversed these effects in hTGF-*β* stimulated HK-2 cells. Similar effects were observed in HK-2 cells with overexpression and with knockdown of MIR6812. We conclude that circMTND5 alleviates renal mitochondrial injury and kidney fibrosis by sponging MIR6812 in LN.

## 1. Introduction

Lupus nephritis (LN) is a major complication of systemic lupus erythematosus (SLE) and affects up to 82% of SLE patients; as many as 44% of LN patients progress to end-stage kidney disease over a period of 15 years [1, 2]. Developing a deeper understanding of the mechanisms of LN and exploring novel therapeutic targets may slow and possibly halt the progression of LN.

Interest in circRNA is expanding rapidly in 2013; two studies on the biogenesis and functions of circRNAs brought these molecules to wider attention [3, 4]. The circBase database for circular RNAs currently lists hundreds of human circRNAs [5]. The genes encoding circRNAs are located in both nuclear and mitochondrial genomes. In the past years, circRNAs have been reported to contribute to the pathogenesis of diverse renal diseases, including renal cell carcinoma, acute kidney injury (AKI), hypertensive nephropathy, diabetic nephropathy, and LN [6, 7]. We previously described upregulation of circHLA-C in renal biopsies from LN patients and suggested that this molecule might contribute to pathogenesis [8]. Subsequently, Zhang et al. have reported that hsa_circ_0123190 acts as a competitive endogenous RNA to sponge miR-483-3p in renal tissues from three untreated LN patients [9]. Other groups reported that hsa_circ_0012919 sponges miR-125a-3p and circIBTK sponges miR-29b in peripheral blood mononuclear cells (PBMCs) from lupus patients [10, 11]. Studies of circRNA in LN have focused on discovering novel biomarkers. Homo sapiens (Hsa) circ_0049224, circ_0049220, and circPTPN22 in PBMCs are associated with SLE activity or severity [12, 13]. Plasma circRNA_002453 may serve as a diagnostic biomarker in LN [14]. These kidney disease-related circRNAs are all nuclear encoded. To date, there has been no report exploring the role of mitochondrial genome-encoded circRNAs in nontumor kidney diseases.

Mitochondrial dysfunction recently has been recognized an important contributor to the pathogenesis of SLE [15, 16]. Mitochondrial uncoupling protein 2- (UCP2-) deficient mice show severe renal mitochondrial fragmentation after ischemia/reperfusion-induced AKI [17]. Overexpression of peroxisomal proliferator-activated receptor gamma-coactivator (PGC-1*α*) can maintain mitochondrial hemostasis of renal tubular cells [18]. Mitochondrial injury can increase the production of reactive oxygen species, which may accelerate cell injury, and promote accumulation of the extracellular matrix, including fibronectin (FN) and collagens (COL), eventuating in progressive fibrosis [19]. Renal fibrosis is a common pathological feature in advanced LN [20]. It remains unknown whether circRNAs influence mitochondrial function and contribute to renal fibrogenesis in LN.

In this study, we aimed to determine whether mitochondrial circMTND5 contributes to the pathogenesis of LN in patients. We identified circMTND5 as the most downregulated circRNA by deep sequencing in LN renal biopsies. We verified that circMTND5 is located in mitochondria and participates in renal mitochondrial injury and kidney fibrosis by sponging MIR6812.

## 2. Materials and Methods

### 2.1. Human Kidney Tissue Samples

Fourteen LN patients with proliferative glomerulonephritis were prospectively enrolled in a clinical study between January 2016 and January 2019 at the department of nephrology of the Affiliated Hospital of China Medical University (Table S1). Twelve renal tumor patients, seen in the department of urology, served as the control group (Table S2). Fresh, unfixed cortical renal biopsy tissues were obtained from LN patients before initiation of steroid and immunosuppressive treatment. Similar normal control cortical kidney tissues were obtained from renal tumor patients; these tissues were located at least 5 cm from the renal tumor. Normal control tissues were stained with periodic acid-Schiff (PAS) and confirmed to be histologically normal by two nephropathologists. Kidney tissues were stored at −80°C until RNA extraction as described previously [8]. A human subject research protocol was approved in advance by the institutional review boards of Shengjing Hospital of China Medical University (15052111). All subjects provided written informed consent prior to research participation.

### 2.2. HK-2 Cell Line and HEK293T Cell Line

The HK-2 cell line, a well-characterized human renal tubular epithelial cell line, and HEK293T cells were purchased from ATCC (Manassas, VA, USA). Cells were cultured in growth medium, DMEM/F-12 or RPMI 1640 medium supplemented with 100 U/mL penicillin G, 100 *μ*g/mL streptomycin, and 10% bovine calf serum at 37°C in a humid atmosphere of 95% air and 5% CO_2_.

### 2.3. Analysis of circRNA Profiling

RNA library preparation and circRNA sequencing were performed by CloudSeq Biotech (Shanghai, China). The rRNA and line RNA-depleted RNA were used to construct RNA libraries with the TruSeq Stranded Total RNA Library Prep Kit (Illumina, CA, USA) according to the manufacturer's instructions. RNA libraries were denatured as single-stranded DNA molecules. The cDNAs were captured on Illumina Flow Cells (Illumina, CA, USA), amplified in situ as clusters, and sequenced with 150 bp paired reads on the HiSeq 4000 sequencing system (Illumina, CA, USA). To generate the profiling of differentially expressed circRNAs between LN kidneys and normal control kidneys, the XY scatter plot was analyzed based on the expression levels of all identified circRNAs and the significant difference between LN and control kidneys by Cluster and TreeView software. The binding sites linking circMTND5 and MIR6812 were predicted by TargetScan and miRanda.

### 2.4. Sanger sequencing and RNase R digestion

Total RNA from HK-2 cells was amplified by PCR, and then, PCR products were analyzed by Sanger sequencing. In additional experiment, total RNA from HK-2 cells was incubated without or with RNase R digestion (Epicentre, Madison, WI, USA) for 30 minutes at 37°C. The relative levels of circMTND5 and GAPDH were assayed by qPCR, normalizing to those measured in the Mock group.

### 2.5. Fluorescence In Situ Hybridization

Fluorescence in situ hybridization (FISH) was performed in human kidney tissues and HK-2 cells following the protocol from the manufacturer. Paraffin-embedded human kidney tissue sections were cut at 4 *μ*m thickness. Sections were deparaffinized, rehydrated, and digested with trypsin at 37°C for 30 min. The slides of kidney sections or cultured HK-2 cells were hybridized with a digoxigenin-horseradish peroxidase- (DIG-HRP-) labeled oligonucleotide probe complementary to circMTND5 or MIR6812 (Table S4) at 37°C overnight followed by incubation with anti-DIG-HRP (Servicebio, Wuhan, China) for 50 min, fluorescein isothiocyanate-tyramide signal amplification for 5 min, and diamidino-phenyl indole (DAPI) to stain DNA for 5 min. Images were captured by immunofluorescence microscopy (Nikon, Tokyo, Japan).

The extent of the hybridization signal was semiquantified as previously reported by Huang et al. [21].

### 2.6. Electron Microscopy

Human kidney tissues were cut into pieces of ~1 mm^3^ size, transferred to 4% paraformaldehyde, post-fixed in 1% osmium tetroxide, dehydrated in graded alcohols, and embedded in Epon (Sigma-Aldrich, MO, USA). Semithin sections were cut in order to confirm that the tissue orientation was satisfactory under the light microscope. Ultrathin sections (30–60 nm) were obtained using a Leica Ultracut UC6 ultramicrotome (Leica Microsystems, Vienna, Austria), mounted on Formvar-coated copper grids, stained with uranyl acetate and lead citrate, and examined using a JEM-1400 digital electron microscopy (JEOL, Tokyo, Japan). Mitochondria injuries were captured at magnification of 15000x and 25000x.

### 2.7. Immunofluorescence Staining

Paraffin-embedded human kidney tissues were cut at 2 *μ*m thickness and were deparaffinized and rehydrated. Antigens were retrieved and nonspecific binding was blocked as previously described [22]. Kidney sections and slides with HK-2 cells were incubated with primary antibodies at 4°C overnight, followed by incubation with Alexa-594/Alexa-488 donkey anti-rabbit/anti-mouse IgG (Table S5). After three washes with PBS, slides were mounted with DAPI for 10 min. Images were captured by immunofluorescence (IF) microscopy (Nikon, Tokyo, Japan). IF was quantified by Image-Pro Plus 6.0 (Media cybernetics, MD, USA).

### 2.8. MitoTracker Red Staining

HK-2 cells were cultured for 6 h on the slides that were placed in culture dishes with HK-2 medium until cells were attached. Next, 100 nM MitoTracker Red CMXRos (Invitrogen, Carlsbad, CA) was added in the medium and incubated for 30 min at 37°C (Solarbio, Beijing, China). The medium was removed and the slides were fixed for FISH.

### 2.9. Mitochondria Isolation from HK-2 Cells

HK-2 cells were homogenized by lysis buffer (Solarbio, Beijing, China) and ground 30 times on ice. The homogenate was transferred to a 1.5 mL of a microfuge tube. The tube was centrifuged at 1000 g for 5 min at 4°C. The supernatant was transferred to a fresh tube and centrifuged again. The supernatant from second centrifugation was collected and was centrifuged at 12000 g for 10 min at 4°C. The crude mitochondria-containing pellet was suspended in 500 *μ*L wash buffer (Solarbio, Beijing, China) and centrifuged at 12000 g for 10 min to obtain final pellet. This pellet, containing a mitochondria-enriched fraction and cytosolic fraction from the final spin, were used for extraction of total RNA. The expression levels of circMTND5 and MIR6812 were measured in mitochondrial fractions by qPCR.

### 2.10. RNA Immunoprecipitation Assay

HK-2 cells or their mitochondria fraction was homogenized in RNA immunoprecipitation (RIP) lysis buffer (Sigma-Aldrich, MO, USA). Antibodies against immunoglobulin G (Anti-IgG) or Argonaute 2(AGO2) conjugated with magnetic beads (Sigma-Aldrich, MO, USA) were incubated with cell lysates overnight at 4°C. Enrichment of circMTND5 in the immunoprecipitation with anti-AGO2 or anti-IgG was measured by qPCR.

### 2.11. RNA Pulldown

The biotinylated circMTND5 or its negative control (Table S4) was transfected into HK-2 cells. The cells were lysed using lysis buffer at 48 h following transfection. Streptavidin agarose beads (Invitrogen, CA, USA) were incubated with the cell lysates, and the RNA complex that was bound to the beads was eluted and purified using TRIzol. qPCR was performed to measure the MIR6812 copy number in the RNA complexes.

### 2.12. Luciferase Reporter Assay

The wide-type binding sites of circMTND5/UCP2 were inserted into the pmirGLO Dual-Luciferase miRNA Target Expression Vector (Promega, CA, USA). The mutated binding site sequences of circMTND5/UCP2 were generated by CloudSeq Biotech (Shanghai, China). Wild-type or mutated circMTND5/UCP2 was cotransfected with MIR6812 mimic/negative control into HEK293T cells. After transfection for 48 h, cells were harvested and the luciferase activity was measured using the Dual-Luciferase Reporter Gene Assay Kit in a luciferase reporter system (Promega, CA, USA).

### 2.13. Knockdown and Overexpression of circMTND5 or MIR6812

To knockdown expression of circMTND5, the cultured HK-2 cells were transfected with circMTND5 RNA interference (RNAi)/negative control or MIR6812 mimic/negative control (SyngenTech, Beijing, China) (Table S3) using Lipofectamine 3000 (Invitrogen, CA) for 24 h according to the manufacturer's instructions. On the other hand, the cultured HK-2 cells were stimulated with human transforming growth factor *β* (hTGF-*β*, 250 pg/mL) for 24 h to downregulate circMTND5 expression. Then, the cells were transfected with one of two pairs of vectors: (1) circMTND5 containing vector (pCDH-CMV-5′ Circular Frame-circMTND5-3′ Circular frame-EF1-copGFP-T2A-Puro) and its corresponding empty vector (pCDH-CMV-5′ Circular Frame-MCS-3′ Circular frame-EF1-copGFP-T2A-Puro) (SyngenTech, Beijing, China) and (2) MIR6812 inhibitor/its negative control (SyngenTech, Beijing, China). Transfections were accomplished using lipofectamine 3000 and/or P3000 (Invitrogen, CA, USA) for 24 h. Cells were collected for RNA and protein extraction 24 h after the transfection of specific genes for examination of qPCR and Western blotting.

### 2.14. Quantitative PCR

Total RNA (250 ng per sample) from kidney tissues and from HK-2 cells was subjected to reverse transcription using the PrimeScript RT Reagent Kit or TransScript miRNA First-Strand cDNA Synthesis SuperMix (TransGen Biotech, Beijing, China) followed by PCR with SYBR Premix Ex Taq (TaKaRa, Dalian, China). Primers were designed using Primer Express (Applied Biosystems, CA, USA) and synthesized by Life Technologies (Shanghai, China) (Table S3). Real-time fluorescence was detected with QuantStudio 6 Flex quantitative PCR system (Applied Biosystems, CA, USA).

### 2.15. Western Blotting

HK-2 cells were homogenized in RIPA buffer with protease inhibitor cocktail. An equal amount of individual protein was separated by SDS-PAGE, and the gels were transferred to PVDF membranes (Millipore-Sigma, MA, USA). After blocking with 5% milk, membranes were incubated with primary antibody (Table S5) overnight at 4°C. The blots were incubated with peroxidase-conjugated goat anti-rabbit/mouse IgG for 60 min at room temperature. The antibody-antigen reactions were detected by High-sig ECL Western Blotting Substrate and visualized by the Tanon 5500 imaging system (Tanon Science and Technology, Shanghai, China). Blot densities were analyzed using ImageJ software (NIH, MD, USA).

### 2.16. Statistical Analysis

GraphPad Prism 9 (GraphPad, San Diego, CA, USA) was used for statistical analysis and graphing. Quantitative data were expressed as mean ± SD. Differences between groups were analyzed for statistical significance by one or two-way ANOVA or *t*-tests. A *p* value < 0.05 was accepted as statistically significant.

## 3. Results

### 3.1. circMTND5 was Downregulated in Renal Biopsies of LN Patients

**Figure 1:**
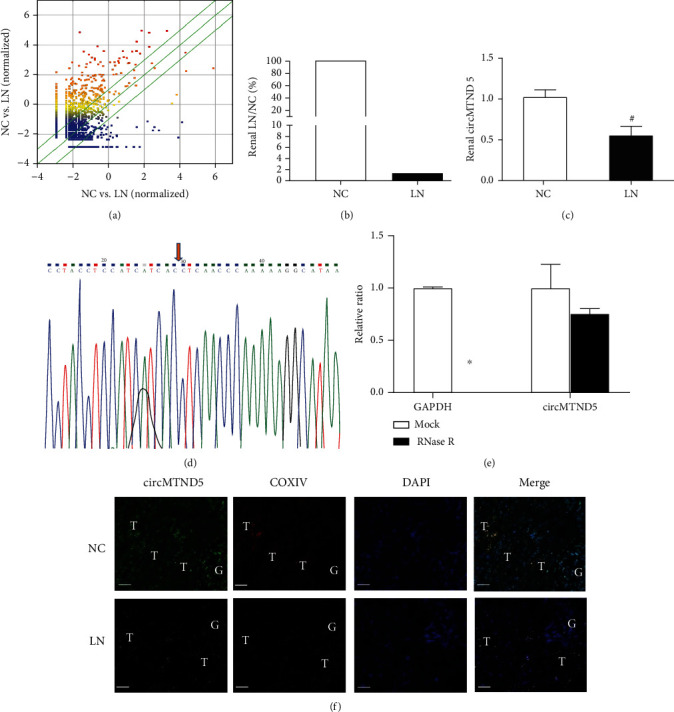
The profiling of circRNAs and downregulation of circMTND5 in renal biopsies from lupus nephritis patients. (a) XY scatter plot. The circRNAs above the top green line and below the bottom green line indicate more than 2.0-fold changes (increased or decreased) in renal biopsies from patients with lupus nephritis compared to normal control kidney tissues. The downregulation of renal circMTND5 on (b) microarray and on (c) validation by qPCR in kidney tissues from lupus nephritis patients and normal controls. (d) Validation of circMTND5 junction sequence by Sanger sequencing. (e) Validation of circMTND5 stability by RNase R digestion in HK-2 cells. (f) Colocalization of mitochondrial COX IV by IF staining and circMTND5 by FISH (magnification 400x, bar = 40 *μ*m. G: glomeruli, T: renal tubules). Data are presented as mean ± SD, ^#^*p* < 0.05, lupus nephritis (*n* = 7) vs. normal controls (*n* = 6). ^∗^*p* < 0.05, RNase R vs. Mock.

The profiling data of renal circRNAs in these biopsies and normal control kidneys can be found at the following two websites: https://www.ncbi.nlm.nih.gov/geo/query/acc.cgi?acc=GSE108340 and https://www.ncbi.nlm.nih.gov/geo/query/acc.cgi?acc=GSE108735 [8]. In total, 168 differentially expressed circRNAs were identified between LN renal biopsies and normal kidney tissues. 136 circRNAs were significantly upregulated and 32 circRNAs were significantly downregulated in LN biopsies compared to normal control kidney tissues (fold change ≥ 2.0 and *p* < 0.05) on the XY scatter plot (Figure 1(a)). We focused on the most downregulated renal circRNA from the list of 32 decreased circRNAs (Table S6). This is a novel human circRNA that we have termed “circMTND5” (Figure 1(b)), with a decrease to 1.4% of the normal control value. The circRNA gene is located on the tin mitochondrial genome (chrM: 14068-14413+), and the host gene is MTND5, encoding the mitochondrially encoded NADH: ubiquinone oxidoreductase.

Next, we validated circMTND5 in different sets of kidney tissue samples by qPCR. circMTND5 was also decreased on qPCR (Figure 1(c)) as that on microarray. To validate this novel mitochondrial circMTND5 as a real circRNA, Sanger sequencing and RNase R digestion were applied to HK-2 cells. The junction sequence of circMTND5 back splicing was ACCT validated by Sanger sequencing (Figure 1(d)). In addition, the expression of circMTND5 decreased about 10% of HK-2 cells treated with Mock after RNase R digestion (Figure 1(e)).

To verify the distribution and expression changes of circMTND5 in renal biopsies from LN patients, FISH showed circMTND5 mainly located in cytoplasm of renal tubular epithelial cells. Further, circMTND5 was colocalized with mitochondrial cytochrome c oxidase subunit 4 (COX IV), a mitochondria-specific endogenous biomarker by double staining of FISH and IF staining. In addition, circMTND5 expression was decreased in the LN kidney compared to the normal kidney (Figure 1(f)).

### 3.2. Mitochondrial Injury and Renal Fibrosis Were Seen in Renal Biopsies from LN Patients

**Figure 2:**
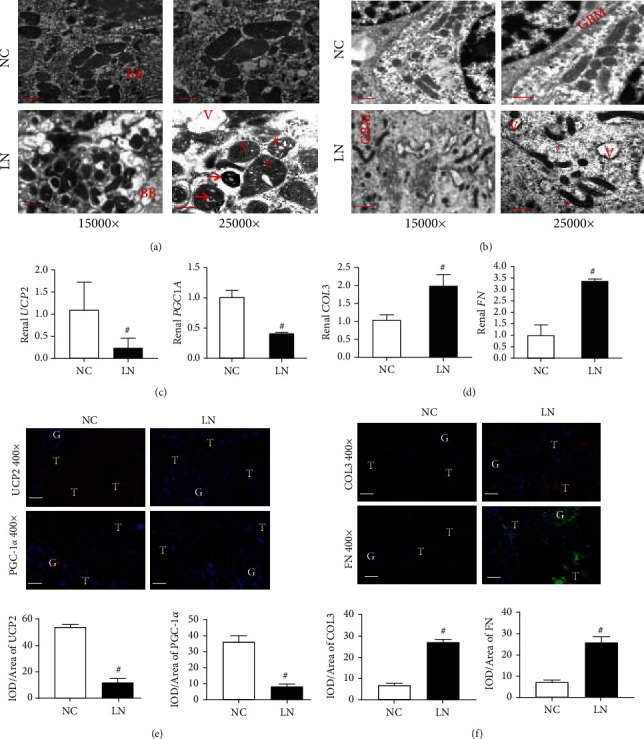
Mitochondrial injury and renal fibrosis in renal biopsies from lupus nephritis patients. (a) Renal tubular cells on electron microscopy. (b) Glomerular podocytes on electron microscopy. (c) mRNA expression of mitochondrial *UCP2* and *PGC1A* genes. (d) mRNA expression of profibrotic *COL3* and *FN* genes. IF staining and semiquantitative analysis of (e) mitochondrial UCP2 and PGC-1*α* as well as (f) profibrotic COL3 and FN. Magnification 15000x, bar = 0.83 *μ*m; magnification 25000x, bar = 0.5 *μ*m. Magnification 400x, bar = 80 *μ*m. TBM: tubular basement membrane; BB: brush border; GBM: glomerular basement membrane; FP: podocyte foot process; V: vacuolization; S: swollen mitochondria; ^∗^shrunken mitochondria; ^#^swollen mitochondria with vacuolization; arrow: mitochondrial autophagy bodies; G: glomeruli; T: tubules; IOD: integrated optical density. Data are presented as mean ± SD, ^#^*p* < 0.05 lupus nephritis (*n* = 7) vs. normal controls (*n* = 6).

We examined morphological changes of mitochondria in renal biopsies from LN patients using electron microscopy (EM). Various morphological features of mitochondrial damages including vacuolization, swollen, shrinking, and autophagy bodies were seen in renal tubular cells and glomerular podocytes in LN renal biopsies on EM under different magnification (Figures 2(a)–2(b)). In addition to morphologic evidence of mitochondria injury seen on EM, we found downregulation of mRNA encoding certain mitochondrial functional genes, including *UCP2* and *PGC1A* in LN renal biopsies compared to normal control kidney tissues on qPCR (Figure 2(c)). Expression of the above two key proteins involved in mitochondria bioenergetics control was also decreased on the IF staining and their semiquantifications (Figure 2(e)). The expression of profibrotic genes, including *COL3* and *FN*, was upregulated in LN kidneys compared to normal kidneys (Figure 2(d)). Similar changes were seen on the IF staining of these two profibrotic proteins and on their semiquantifications (Figure 2(f)).

### 3.3. circMTND5 Localized in Mitochondria

Since the gene encoding circMTND5 is located in the mitochondrial genome, we verified the localization of circMTND5 by three approaches. First, we demonstrated colocalization of MitoTracker Red staining and circMTND5 by FISH in cytoplasm of HK-2 cells (Figure S1A). Second, we showed colocalization of circMTND5 RNA and the mitochondrion-specific protein COX IV by double staining of FISH and IF staining (Figure S1B). Third, using isolated mitochondrial fraction from HK-2 cells, we confirmed that the circMTND5 level was increased 86-fold in the mitochondrial fraction compared to the cytosolic fraction lacking mitochondria (Figure S1C).

### 3.4. circMTND5 Served as a Sponge of MIR6812

**Figure 3:**
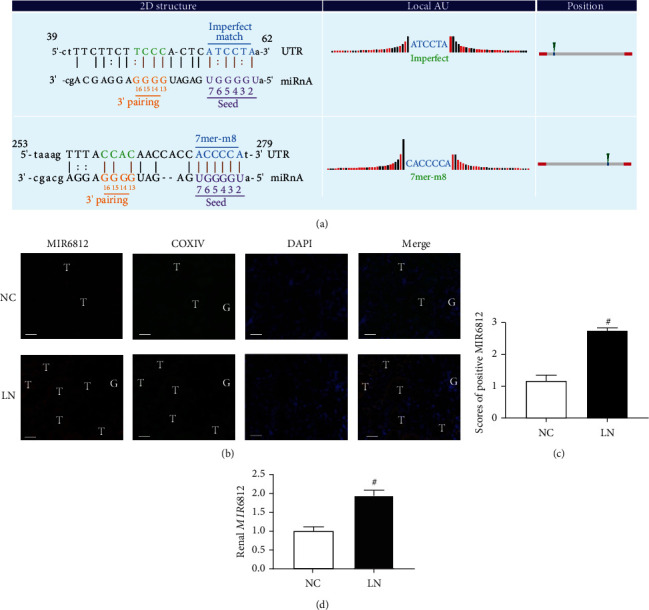
Expression of MIR6812 in human kidney tissues. (a) Binding site sequences between circMTND5 and MIR6812 as predicted by the applications TargetScan and miRanda. (b, c) Colocalization of mitochondrial COX IV by IF staining and MIR6812 by FISH and semiquantification of FISH for MIR6812 expression in kidney (magnification 400x, bar = 40 *μ*m. G: glomeruli; T: renal tubules). (d) Renal expression of MIR6812 on qPCR. Data are presented as mean ± SD; ^#^*p* < 0.05 lupus nephritis (*n* = 7) vs. normal controls (*n* = 6).

Studies of the function of circRNAs have been focused on their roles in sponging miRNAs [23]. TargetScan and miRanda predicted the binding site sequences between circMTND5 and its top five microRNAs including MIR6812 (Figure S2). Further bioinformatics analysis found that circMTND5 and MIR6812 had two binding site sequences (Figure 3(a)). The renal expression of MIR6812 was increased in LN renal biopsies compared to normal kidney tissues on FISH staining (Figures 3(b) and 3(c)) and qPCR assay (Figure 3(d)). In addition, MIR6812 colocalized with COX IV in mitochondria of renal tubular cells of human kidney tissues (Figure 3(b)).

**Figure 4:**
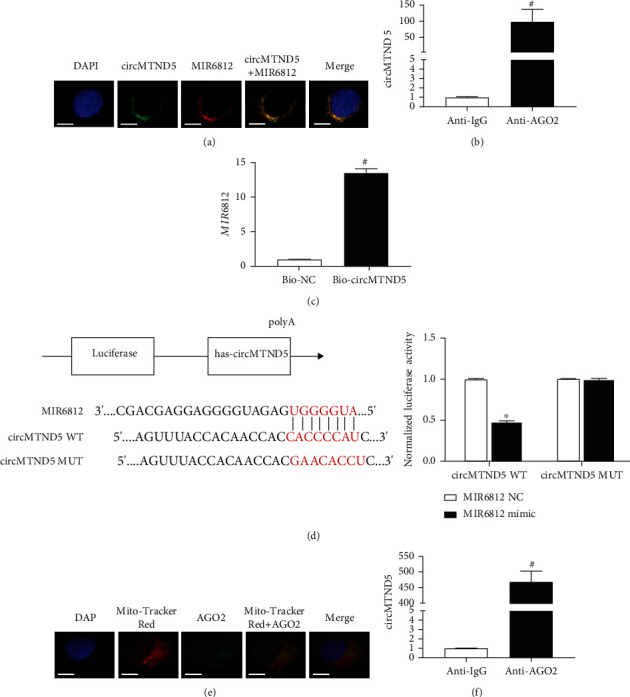
Interaction between circMTND5 and MIR6812 in HK-2 cells. (a) Colocalization of circMTND5 and MIR6812 by double FISH (magnification 725x, bar = 80 *μ*m). (b) Relative enrichment of circMTND5 demonstrated in HK-2 cells using RIP with anti-AGO2 antibody compared to anti-normal IgG. (c) Overexpression of MIR6812 in HK-2 cells with transfection of biotinylated circMTND5 or biotinylated negative control using RNA pulldown assay. (d) The luciferase activity of circMTND5 in HEK293T cells with cotransfection of MIR6812 mimic and circMTND5 WT or circMTND5 MUT. (e) Double staining using MitoTracker Red and anti-AGO2 antibody (magnification 725x, bar = 80 *μ*m). (f) Relative enrichment of circMTND5 using RIP with anti-AGO2 antibody compared to anti-normal IgG in mitochondrial fraction of HK-2 cells. Data are presented as mean ± SD of three experiments, each with triplicate samples. ^#^*p* < 0.05 anti-AGO2 vs. anti-IgG or Bio-circMTND5 vs. Bio-NC or mitochondrial fraction vs. cytosolic fraction. ^∗^*p* < 0.05 MIR6812 mimic vs. MIR6812 negative control.

After, the colocalization of circMTND5 and MIR6812 was found in the cytosol of HK-2 cells on double staining of FISH (Figure 4(a)). A standard set of RIP, RNA pulldown, and luciferase activity assay was performed to verify the interaction between circMTND5 and MIR6812. circMTND5 levels were significantly increased 86-fold in HK-2 cells exposed to AGO2 antibody compared to cells exposed to nonspecific IgG (Figure 4(b)). MIR6812 expression was increased 13.5-fold in HK-2 cells transfected with the bio-circMTND5 compared to the negative control (Figure 4(c)). With cotransfection of MIR6812 mimic and wild-type DNA sequence encoding circMTND5 (circMTND5 WT) or a mutated sequence encoding circMTND5 (circMTND5 MUT) into HEK293T cells, luciferase reporter gene assay was performed. Luciferase activity was decreased to 47% of the baseline with circMTND5 WT and MIR6812 mimic compared to the cells receiving circMTND5 MUT plus MIR6812 mimic (Figure 4(d)).

The next question that we clarified is that how mitochondrial-derived circMTND5 sponge MIR6812 and affect mRNA. We first investigated whether AGO2 also localizes in mitochondria. The colocalization of MitoTracker Red and AGO2 was shown in cytosols of HK-2 cells on the costaining (Figure 4(e)). Further RIP experiment demonstrated that the circMTND5 level was increased much higher in the mitochondrial fraction than in whole cells exposed to AGO2 antibody compared to the cells exposed to normal IgG antibody (Figure 4(f)).

### 3.5. circMTND5 Ameliorates Mitochondrial Injury and Fibrosis by Sponging MIR6812

**Figure 5:**
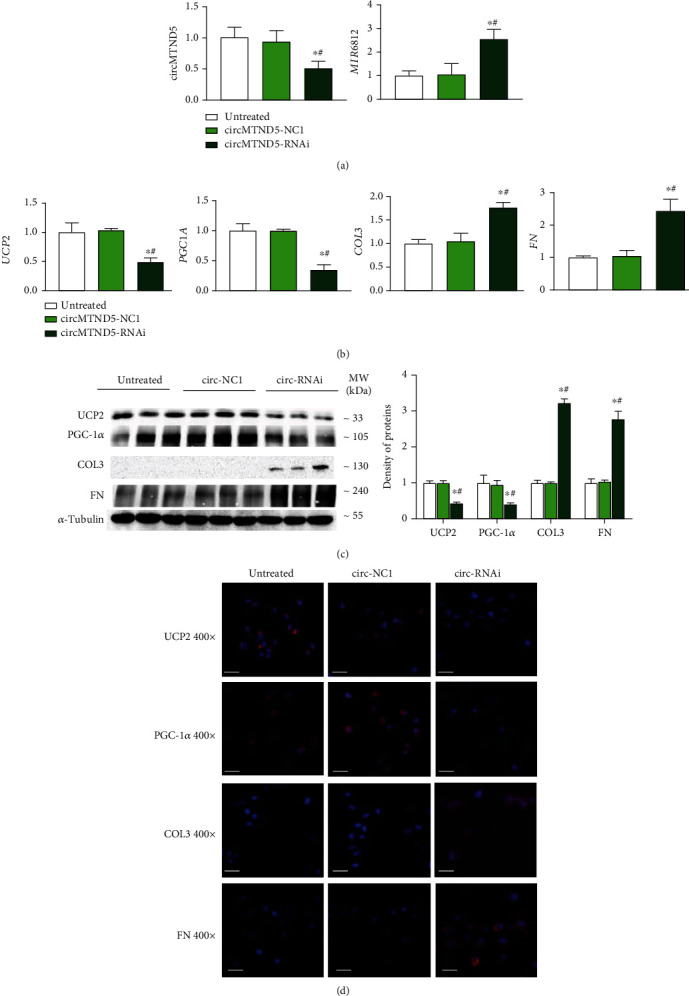
Knockdown of circMTND5 in HK-2 cells. (a) Expression of circMTND5 and MIR6812 post-circMTND5 knockdown in normal HK-2 cells. (b) Expression of mitochondrial UCP2 and PGC-1*α* as well as profibrotic COL3 and FN post-circMTND5 knockdown on the mRNA level. Expression of mitochondrial UCP2 and PGC-1*α* as well as profibrotic COL3 and FN post-circMTND5 knockdown on protein by (c) Western blotting and (d) IF staining (magnification 400x, bar = 40 *μ*m). Data are presented as mean ± SD from three experiments, each with triplicate samples. ^∗^*p* < 0.05 each group vs. the untreated group; ^#^*p* < 0.05 circMTND5 RNAi vs. circMTND5 NC1.

**Figure 6:**
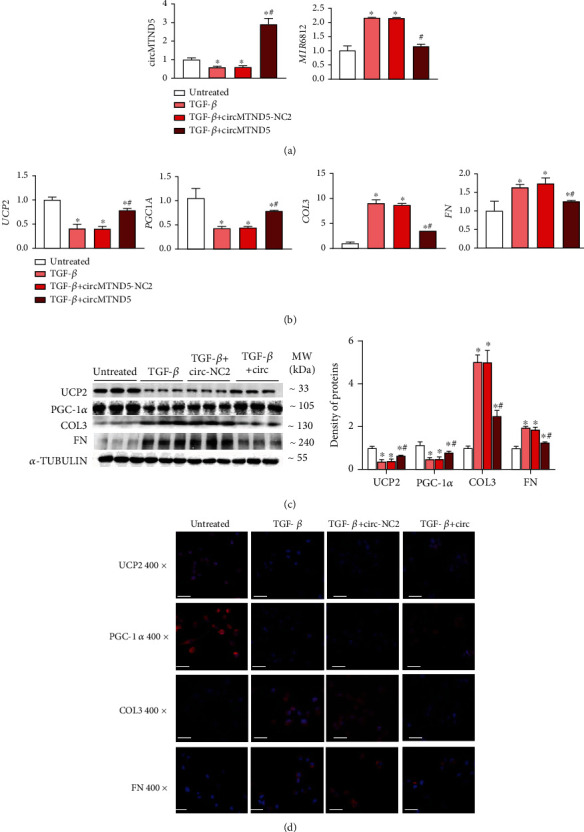
Overexpression of circMTND5 in HK-2 cells with hTGF-*β* stimulation. (a) Expression of circMTND5 and MIR6812 following overexpression of circMTND5 in HK-2 cells with hTGF-*β* stimulation. (b) Effects of circMTND5 overexpression on the expression of mitochondrial genes encoding UCP2 and PGC-1*α* and profibrotic *COL3* and *FN* genes, at the mRNA level. Effects of circMTND5 overexpression on the expression of mitochondrial genes encoding UCP2 and PGC-1*α* as well as profibrotic *COL3* and *FN* genes on the protein level evaluated by (c) Western blotting and (d) IF staining (magnification 400x, bar = 40 *μ*m). Data are presented as mean ± SD from three experiments, each with triplicate samples. ^∗^*p* < 0.05 each group vs. untreated group; ^#^*p* < 0.05 circMTND5 vs. circMTND5 NC2.

We next sought to determine the effect of circMTND5 on MIR6812, mitochondrial injury, and cellular fibrosis. We performed *knockdown* and *overexpression* experiments of circMTND5 in HK-2 cells and in HK-2 cells with hTGF-*β*-induced decrease of circMTND5. Knockdown of circMTND5 in HK-2 cells upregulated the expression of MIR6812 (Figure 5(a)). In addition, perfect binding sites were also predicted between MIR6812 and mitochondrial inner-membrane-localized gene UCP2, and direct interaction of MIR6812 and UCP2 was confirmed through luciferase reporter assays (Figure S3). The downregulation of mitochondrial UCP2 and PGC-1*α* and upregulation of profibrotic COL3 and FN genes were confirmed on both mRNA by qPCR analysis (Figure 5(b)) and protein levels by Western blot and IF staining (Figures 5(c) and 5(d)). In contrast, *overexpression* of circMTND5 in HK-2 cells with hTGF-*β*-induced decrease of circMTND5 reversed three sets of effects from hTGF-*β* stimulation including the upregulation of MIR6812, downregulation mRNA and protein levels of UCP2 and PGC-1*α*, and the increased expression of COL3 and FN on qPCR, Western blot, and IF staining (Figure 6).

### 3.6. MIR6812 Aggravates Mitochondrial Injury and Fibrosis via Decreasing UCP2

**Figure 7:**
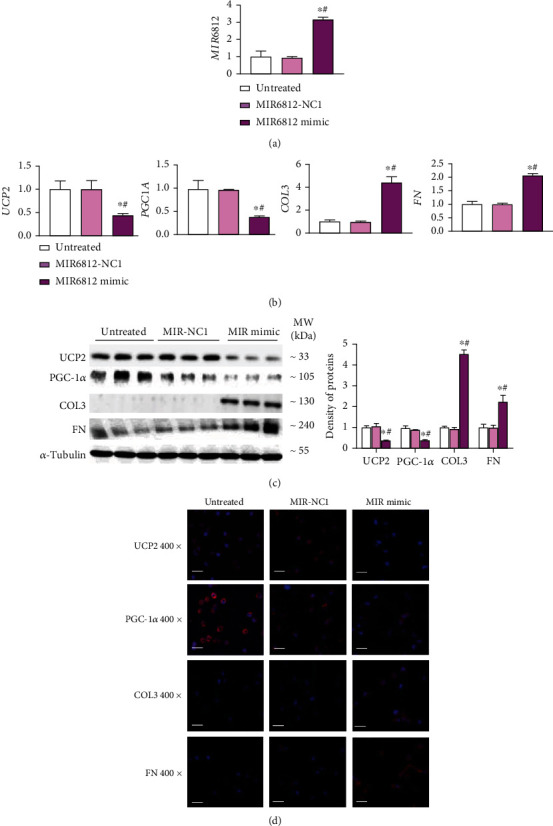
Overexpression of MIR6812 in HK-2 cells. (a) Overexpression of MIR6812 by MIR6812 mimic transfection to normal HK-2 cells. (b) mRNA expression of mitochondrial UCP2 and PGC-1*α* as well as the profibrotic proteins COL3 and FN following MIR6812 mimic transfection. (c-d) Protein expression of mitochondrial UCP2 and PGC-1*α* as well as profibrotic protein COL3 and FN on (c) Western blotting and (d) IF staining (magnification 400x, bar = 40 *μ*m) following MIR6812 mimic transfection. Data are presented as mean ± SD from three experiments, each with triplicate samples. ^∗^*p* < 0.05 each group vs. the untreated group; ^#^*p* < 0.05 MIR6812 mimic vs. MIR6812 NC1.

**Figure 8:**
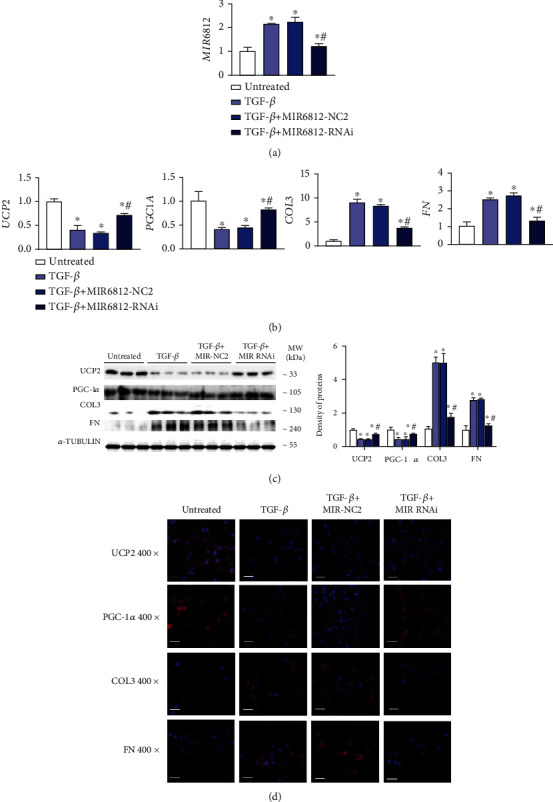
Knockdown of MIR6812 in HK-2 cells. (a) Increased expression of MIR6812 by hTGF-*β* stimulation in normal HK-2 cells. MIR6812 inhibiter reversed overexpression of MIR6812 induced by hTGF-*β*. (b) hTGF-*β*-induced mRNA expression changes of mitochondrial UCP2 and PGC-1*α* as well as profibrotic COL3 and FN were reversed by the MIR6812 inhibitor on mRNA. (c-d) hTGF-*β*-induced protein expression changes of mitochondrial UCP2 and PGC-1*α* as well as profibrotic COL3 and FN were reversed by the MIR6812 inhibitor on (c) Western blotting and (d) IF staining (magnification 400x, bar = 40 *μ*m). Data are presented as mean ± SD from three experiments, each with triplicate samples. ^∗^*p* < 0.05 each group vs. the untreated group, ^#^*p* < 0.05 MIR6812 RNAi vs. respective MIR6812 NC2.

After confirming the effect of the circMTND5 on MIR6812, mitochondrial injury, and cellular fibrosis, we further investigated the direct effect of a MIR6812 mimic and an inhibitor on mitochondrial function and fibrogenesis. Overexpression of MIR6812 was seen in HK-2 cells following MIR6812 mimic transfection (Figure 7(a)). The downregulation of UCP2 and PGC-1*α* genes and the upregulation of profibrotic COL3 and FN genes were found at both mRNA and protein levels (Figures 7(b)–7(d)). The direct effect of the MIR6812 mimic on gene expression was similar to the effect of circMTND5 knockdown. On the other hand, the MIR6812 inhibitor also reversed hTGF-*β*-induced downregulation of mitochondrial functional UCP2 and PGC-1*α* gene as well as the upregulation of profibrotic COL3 and FN compared to their respective negative control on qPCR, Western blot, and IF staining (Figure 8).

## 4. Discussion

**Figure 9:**
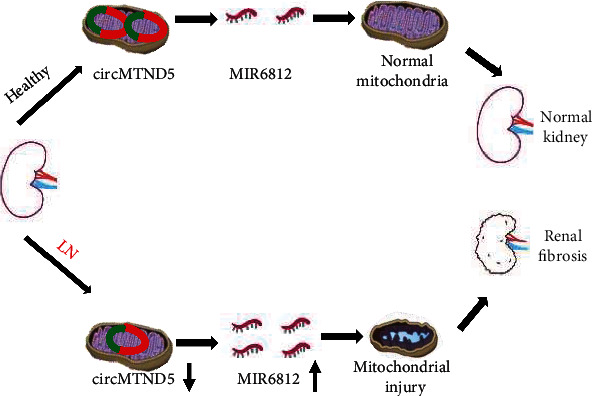
Schematic mechanism model of circMTND5 in renal mitochondrial injury and fibrosis in LN. circMTND5 participates in renal mitochondrial injury and fibrosis in lupus nephritis by sponging MIR6812.

The main findings of this study are as follows: (1) circMTND5 localized to mitochondria and was the most downregulated circRNA in LN biopsies, (2) circMTND5 served as a sponge of MIR6812 in human kidney tissues and HK-2 renal tubular cells, and (3) circMTND5/MIR6812/UCP2 pathway participated in renal mitochondrial injury and renal fibrosis in LN (Figure 9).

In the past few years, circRNAs have been reported to play important roles in the pathogenesis of certain renal diseases, including renal cell carcinoma, AKI, diabetic nephropathy, hypertensive nephropathy, and LN [6, 7]. Here, we have identified circHLA-C as the top upregulated circRNA in renal biopsies from LN patients. We also found that renal circHLA-C correlates with proteinuria, renal function, and clinical pathological indices [8]. After our report, two studies showed that hsa_circ_0012919 serves as a sponge of miR-125a-3p in CD4+ T cells and circIBTK can sponge miR-29b in PBMCs from SLE patients [10, 11]. hsa_circ_0049224, has_circ_0049220, and circPTPN22 in PBMCs [12, 13] and plasma circRNA_002453 may serve as potential diagnostic biomarkers for LN [14]. Tian et al. found that circRNA-34428 is the most overexpressed circRNA in lupus mice but this study did not elucidate its functions in renal disease progression [24].

Beyond lupus, circRNAs have been demonstrated to play an important role in other kidney diseases, in animals, and in human beings. Three groups explored the differential expression of circRNA profiles in animal kidney tissue from rats with hypertension or kidney stones and from mice with AKI induced by ischemia/reperfusion or cisplatin [25–28]. Deng et al. reported that silencing the circular ANRIL protected HK-2 cells from lipopolysaccharide-induced inflammatory injury through upregulating miR-9 *in vitro* [29]. We also reported that renal circZNF609 participated in the pathogenesis of focal segmental glomerulosclerosis [30]. circ_0000524 promotes podocyte apoptosis by sponging miR-500a in membranous nephropathy [31]. circRNA_010383 sponging miR-135a, circHIPK3 sponging miR-185, circEIF4G2 sponging miR-218, circ_0000491 sponging miR-455-3p, circLRP6 sponging miR-205, and circ_15698 sponging miR-185 contribute to cell death, extracellular matrix production, or renal fibrosis in diabetic nephropathy [32–37]. Our group reported that circHIPK3 aggravate renal tubulointerstitial fibrosis by regulating miR-30a [38]. In human kidney diseases, circRNAs have been profiled in exosomes isolated from serum and urine samples from patients with idiopathic membranous nephropathy [39]. In AKI patients, circRNA-126 can predict mortality [40]. To date, the circRNAs studied in renal diseases have all been encoded on autosomal chromosomes. The role of mitochondrial-encoded circRNAs in kidney diseases has rarely been reported.

circHLA-C was identified as the top upregulated one in our previous study, but its molecule is too long to transfect into cells, which limits mechanistic studies. In the present study, we found that circMTND5 was the most downregulated circRNA in LN. In addition, circMTND5 is encoded by a mitochondrial gene, it was most expressed on renal tubular cells by FISH staining. Since circMTND5 was identified as a novel circRNA, we identified its back splicing junction sequence by Sanger sequencing and validated its stability by RNase R digestion (Figure 1). Liu et al. found that mitochondrial-encoded circular RNA (mecci) ND1 and mecciND5 facilitate mitochondrial protein importation by serving as molecular chaperones. This study demonstrates that mecciND5 is encoded in the mitochondrial genome, from position 13846 and to 13999, including 153 nucleic acids, and the function as molecular chaperones in HEK293T cells [41]. We investigated the roles of circMTND5 using human kidney tissue and HK-2 cells. The difference between the circMTND5 reported here and mecciND5 described by Liu et al. might be due to differences in human cell types. circRNAs typically manifest tissue-specific and cell type-specific features [23]. In addition, Mance et al. demonstrated that mitochondrial mRNA fragments can be circularized in HEK293T cells [42]. Mitochondrial genome-derived circRNA-COX2, one more mitochondrial circRNA so far, was demonstrated to serve as an oncogene in chronic lymphocytic leukemia [43]. Our data, together with the work of others, suggest that mitochondrial circRNAs may maintain mitochondrial functions via multiple mechanisms.

The kidney is the organ with the second highest mitochondria abundance following the heart [44]. In the kidney, tubular cells contain the highest mitochondrial numbers [45]. We found that various mitochondrial injury features in lupus renal biopsies on EM (Figure 2) with similar findings in lupus mice and CKD rats resulted from AKI [45, 46]. Renal fibrosis is a common pathological feature in kidney biopsies from LN patients [22]. What is the role of circMTND5 in mitochondrial injury and renal fibrosis? UCP2-deficient mice can aggravate kidney mitochondrial injury from AKI [17]. PGC-1*α* is a well-known key regulator of mitochondria biogenesis [47]. PGC-1*α* transgenic mice manifest protection from Notch-induced kidney fibrosis [18]. Consistent with these findings, we found that the expression of UCP2 and PGC-1*α* was decreased in renal biopsies from LN patients compared to normal kidney tissue. Mitochondrial damages can cause renal fibrosis via increasing inflammation [19]. We also found that the expression of renal COL3 and FN was also increased in the kidneys of LN patients (Figure 2). These data suggest that renal downregulation of circMTND5 may contribute to renal mitochondrial injury and fibrosis in LN.

What are the underlying mechanisms of circMTND5 in the pathogenesis of LN? Several studies have demonstrated that circRNAs serve as a sponge of miRNAs to prevent their action [23]. MIR6812 was predicted to have two perfect binding site sequences with circMTND5 by TargetScan and miRanda. Thus, we further examined the expression of MIR6812 in renal biopsies from LN patients by FISH, IF staining, and qPCR. We found that MIR6812 was increased while circMTND5 decreased. In addition, MIR6812 colocalized with COX IV, a mitochondrion-specific endogenous control protein (Figure 3). Since the circMTND5/MIR6812 axis has not been reported in any diseases, we verified the direct interaction between circMTND5 and MIR6812 by double FISH of circMTND5 and MIR6812, RIP assay, and RNA pulldown coupled with luciferase reporter assay. We further confirmed enriched circMTND5 in the mitochondria of renal tubular cells by RIP with AGO2 antibody compared to normal IgG antibody. We also showed the enriched level of circMTND5 in mitochondrial fraction compared to cytosolic fraction in HK-2 cells (Figure 4(f)). This data suggests that circMTND5 mainly sponged MIR6812 in mitochondria. Based on this direct evidence of circMTND5 sponging MIR6812 and the ideal length of circMTND5 including 346 nucleic acids for transfection studies, we further studied the effect of circMTND5 and MIR6812 in HK-2 cells by knockdown and overexpression of two genes.

circMTND5 *knockdown* upregulated MIR6812, decreased expression of mitochondrial functional genes (UCP2 and PGC-1*α*), and increased expression of profibrotic genes (COL3 and FN). The o*verexpression* of circMTND5 reversed these effects in hTGF-*β*-stimulated HK-2 cells on qPCR analysis, Western blot, and IF staining (Figures 5–6). Knockdown and overexpression of circMTND5 displayed the expected changes of MIR6812, mitochondrial genes UCP2, and PGC-1*α* as well as profibrotic genes COL3 and FN. These data suggested that circMTND5 is the trigger factor in the mitochondria injury and fibrosis formation in the development of LN.

Based on the above findings, we further investigate the effect of knockdown and overexpression of MIR6812, one of the top five microRNAs of circMTND5. We found that that MIR6812 directly downregulated mitochondrial UCP2 by luciferase reporter assays though binding 3′UTR of UCP2 (Figure S3). Further, MIR6812 mimic transfection to HK-2 cells downregulated expression of mitochondrial UCP2 and PGC-1*α* gene and upregulated expression of profibrotic COL3 and FN (Figure 7). On the other hand, MIR6812 inhibitor reversed hTGF-*β*-induced downregulation of UCP2 and PGC-1*α* gene and the upregulation of COL3 and FN in HK-2 cells (Figure 8). Taken together, our data suggested that circMTND5 might contribute to mitochondrial injury and further promote renal fibrosis by sponging MIR6812; further, MIR6812 regulate UCP2 and PGC-1*α* to participate renal fibrosis in lupus nephritis.

## 5. Conclusions

circMTND5 may play an important role in improving mitochondrial injury and attenuating renal fibrosis in LN by sponging MIR6812. The interference of the circMTND5/MIR6812 axis may offer potential novel avenues for RNA therapeutics to treat renal mitochondrial injury and renal fibrosis in LN.

## Data Availability

The datasets used and/or analyzed during the current study are available from the corresponding author upon reasonable request.

## Supplementary Materials

Supplementary Materials Supplemental Figure 1: localization of circMTND5 to mitochondria of HK-2 cells. Supplemental Figure 2: the top five miRNAs interacting with circMTND5. Supplemental Figure 3: interaction between MIR6812 and UCP2 in HEK293T cells. Supplemental Table 1: the clinical characteristics of lupus nephritis patients. Supplemental Table 2: the clinical characteristics of normal control human subjects. Supplemental Table 3: RNA sequences used in qPCR and cell transfection. Supplemental Table 4: probes used in FISH and RNA pulldown. Supplemental Table 5: antibodies used in immunostaining and Western blotting. Supplemental Table 6: 32 significantly decreased circRNAs in LN biopsies.
