# Supplementary material for: circMTND5 Participates in Renal Mitochondrial Injury and Fibrosis by Sponging MIR6812 in Lupus Nephritis
Source: Oxid Med Cell Longev. 2022 Oct 11;2022:2769487. doi: 10.1155/2022/2769487 (PMC9578797; doi:10.1155/2022/2769487)
Supplement: Supplementary Materials — Supplemental Figure 1: localization of circMTND5 to mitochondria of HK-2 cells. Supplemental Figure 2: the top five miRNAs interacting with circMTND5. Supplemental Figure 3: interaction between MIR6812 and UCP2 in HEK293T cells. Supplemental Table 1: the clinical characteristics of lupus nephritis patients. Supplemental Table 2: the clinical characteristics of normal control human subjects. Supplemental Table 3: RNA sequences used in qPCR and cell transfection. Supplemental Table 4: probes used in FISH and RNA pulldown. Supplemental Table 5: antibodies used in immunostaining and Western blotting. Supplemental Table 6: 32 significantly decreased circRNAs in LN biopsies. [file 2769487.f1.pdf]

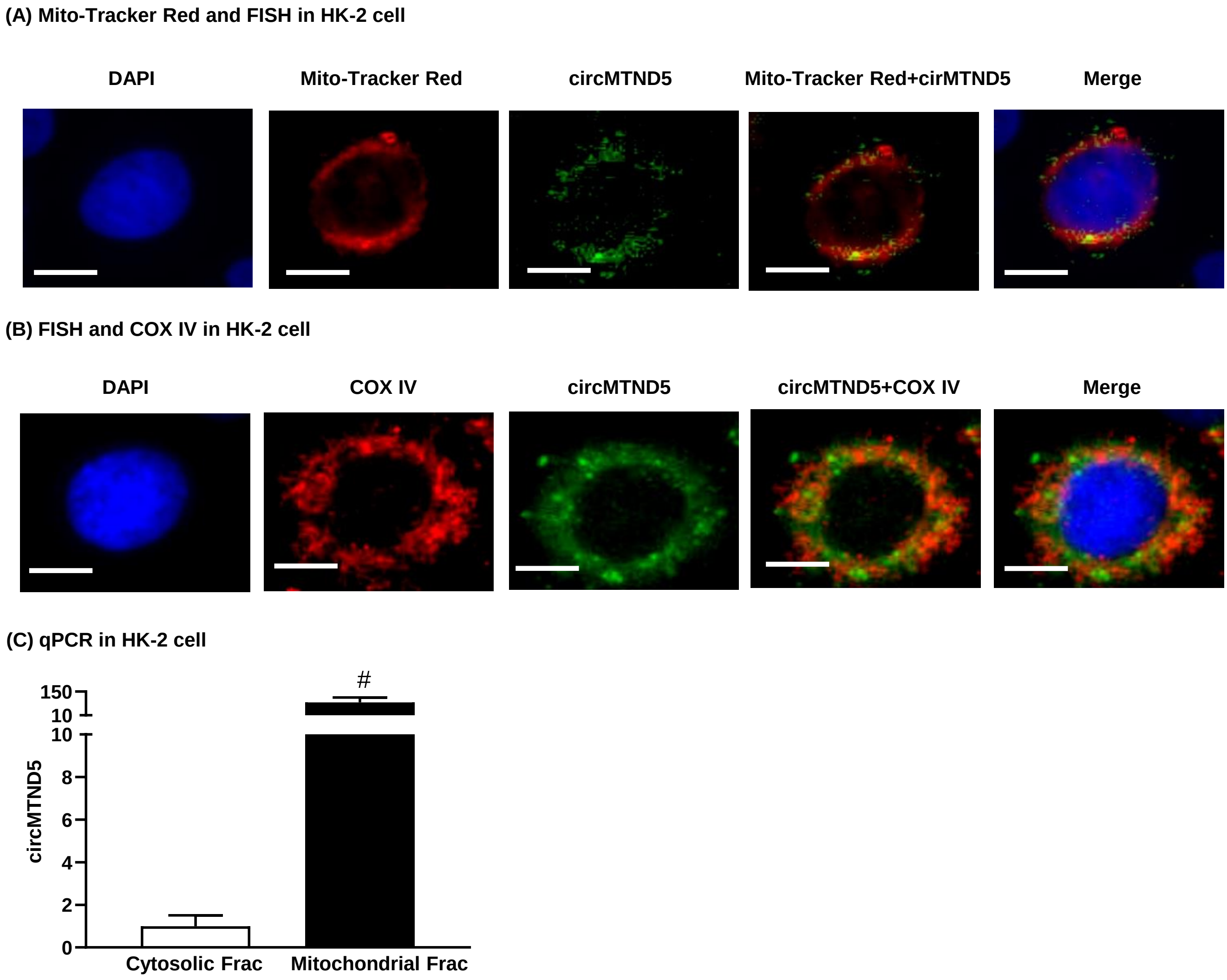

Supplemental Figure 1. Localization of circMTND5 to mitochondria of HK-2 cells. (A) Co-localization of Mito-Tracker Red staining and circMTND5 by FISH demonstrates the circMTND5 is present in mitochondria, arrayed in circle around the nucleus (magnification 725×, bar=80μm). B) Co-localization of mitochondrial cytochrome c oxidase subunit 4 (COX IV) by IF staining and circMTND5 by FISH (magnification 725×, bar=80μm). (C) Expression of circMTND5 in mitochondrial fraction and cytosolic fraction. Data are presented as mean ± SD, from three experiments with triplicate respectively, # denotes $p<0.05$, mitochondrial fraction vs. cytosolic fraction.

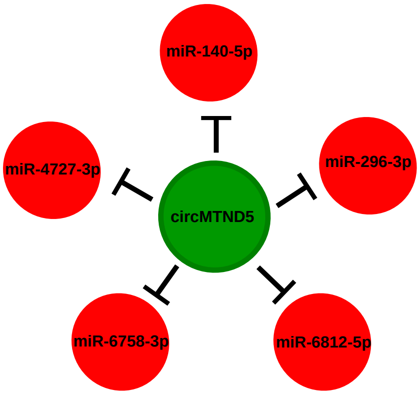

Supplemental Figure 2. The top five miRNAs interacting with circMTND5.
Schematic cartoon of circMTND5 in pathogenesis of LN by sponging MIR6812 and other four miRNAs based on the binding strength.

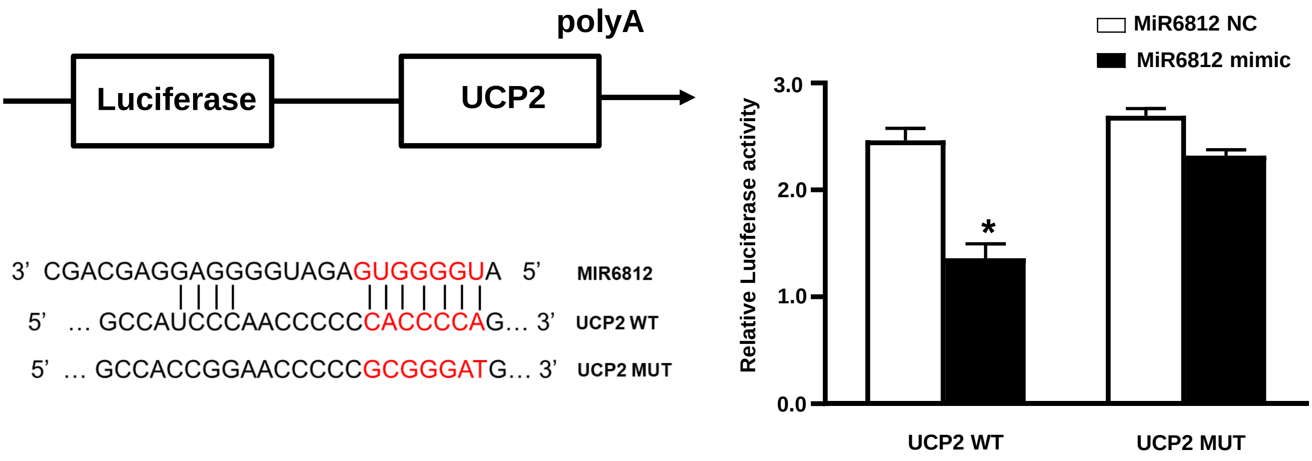

Supplemental Figure 3. Interaction between MIR6812 and UCP2 in HEK293T cells. Direct interaction of MIR6812 and UCP2 was confirmed by luciferase reporter assays

Supplemental Table 1. The clinical characteristics of lupus nephritis patients

Pt. #	Age	Gender	SLEDAI (score)	eGFR (ml/min/1.73m^2)	Urine: URTP (g/day)	Blood: Alb (g/L)	Blood: ANA	Blood: ds-DNA	Blood: IgG (g/L)	Blood: C4 (g/L)	Renal biopsy: AI (score)	Renal biopsy: CI (score)
1	52	F	22	44	5.8	24.3	2+	1+	7.3	0.07	13	5
2	26	F	26	138	0.5	20.1	2+	1+	28.3	0.17	10	0
3	35	F	22	50	2.5	21.5	2+	1+	12.4	0.03	10	3
4	40	F	18	46	15.3	21.0	2+	-	9.3	0.15	13	2
5	40	F	20	120	0.2	19.5	2+	-	19.9	0.02	7	0
6	49	F	21	112	3.5	24.4	2+	1+	12.6	0.03	4	1
7	39	M	25	125.2	10.9	23.2	2+	2+	5.5	0.1	13	0
8	18	F	36	221	8.9	15.8	2+	-	12.6	0.03	1	0
9	36	F	30	123	2.5	28.3	2+	1+	21.7	0.05	4	0
10	44	F	14	94	2.6	28.3	1+	-	18.1	0.25	8	6
11	22	F	22	139	15.8	15.0	2+	2+	7.6	0.07	14	0
12	41	F	17	91	1.49	23.0	2+	±	12.7	0.1	9	1
13	39	F	10	92.75	1.66	32.3	2+	-	13.8	0.15	4	6
14	26	M	18	206.01	6.62	32.8	1+	-	10.8	0.03	13	3

Pt, patient; M/F, male/Female; SLEDAI, systemic lupus erythematous diseases activity index; eGFR, estimated glomerular filtration rate based on CKD-EPI formula; URTP, urinary total proteinuria per day; Alb, albumin; ANA, antinuclear antibodies; ds-DNA, double strands DNA; IgG, immunoglobulin G; C4, complement 4; AI, activity index; CI, chronicity index. #1-7 for circRNA microarray, #8-14 for circRNA validation

Supplemental Table 2. The clinical characteristics of normal control human subjects

NC#	Age	Gender	eGFR (ml/min/1.73m^2)	Urine Proteinuria	Urine Gravity	Serum Alb (g/L)
1	32	F	106	±	1.024	43.6
2	50	F	139	-	1.016	37
3	47	F	86	-	1.029	40.9
4	53	F	122	1+	1.016	42.3
5	53	F	120	-	1.01	41.7
6	60	M	125.1	-	1.019	38.4
7	47	F	117	±	1.025	45.2
8	59	F	115	-	1.009	36.4
9	46	F	104	-	1.016	43
10	54	F	122	-	1.021	40.7
11	52	F	123	±	1.024	40
12	42	M	96.9	-	1.008	47.8

NC, normal control indicated normal kidney tissue from patients with renal tumor. eGFR, estimated glomerular filtration rate based on CKD-EPI formula; Alb, albumin. #1-6 for circRNA microarray, #7-12 for circRNA validation

Supplemental Table 3. RNA sequences used in qPCR and cell transfection

Gene	Forward(5'-3')	Reverse(5'-3')
UCP2	CCCCGAAGCCTCTACAATGG	CTGAGCTTGGAATCGGACCTT
PGC1A	TCTGAGTCTGTATGGAGTGACAT	CCAAGTCGTTCACATCTAGTTCA
OPA1	TGTGAGGTCTGCCAGTCTTTA	TGTCCTTAATTGGGGTCGTTG
COL3	GGAGCTGGCTACTTCTCGC	GGGAACATCCTCCTTCAACAG
FN	CGGTGGCTGTCAGTCAAAG	AAACCTCGGCTTCCTCCATAA
circMTND5	CAACCACCACCCCATCAT	ATTGCTCGGGGGAATAGG
GAPDH	GGAGCGAGATCCCTCCAAAAT	GGCTGTTGTCATACTTCTCATGG
MIR6812	ATGGGGTGAGATGGGGAGGAGCAGC	CAGTGCGTGTCGTGGAGT
U6	GGGATGGGGTGAGATGGGGAGG	CAGTGCGTGTCGTGGAGT
circMTND5-NC1	UUCUCCGAACGUGUCACGUTT	ACGUGACACGUUCGGAGAATT
circMTND5-RNAi	TCACCAAGACCTCAACCCA	
MIR6812-NC1	UCACAACCUCCUAGAAAGAGUAGA	UCUACUCUUUCUAGGAGGUUGUGA
MIR6812 mimic	AUGGGGUGAGAUGGGGAGGAGCAGC	GCUGCUCCUCCCCAUCUCACCCCAU
MIR6812-NC2	UCUACUCUUUCUAGGAGGUUGUGA	
MIR6812 inhibitor	GCUGCUCCUCCCCAUCUCACCCCAU	

UCP2, **uncoupling protein 2;** ***PGC1A,*** **peroxisome proliferator-activated receptor gamma coactivator 1-alpha; OPA1, optic atrophy 1;** ***COL3,*** **collagen type III ;** ***FN,*** **fibronectin. circ-NC1, negative control of circMTND5 RNA interference; circ-RNAi, circMTND5 RNA interference; MIR6812-NC1, negative control of MIR6812 mimic; MIR6812-NC2, negative control of MIR6812 inhibitor.**

Supplemental Table 4. Probes used in FISH and RNA pull-down

Gene	5'-3'	Application
circMTND5-NC	FAM-ACTCACCAAGACCTCAACCCAAAAAGGCA-FAM	FISH
circMTND5	FAM-TTTGGGTTGAGGTCTTGGTGAGTGTT-FAM	FISH
MIR6812-NC	CY3-TCACAACCTCCTAGAAAGAGTAGA-CY3	FISH
MIR6812	CY3-GCTGCTCCTCCCCATCTCACCCCAT–CY3	FISH
miR-150	FAM-CACTGGTACAAGGGTTGGGAGA-FAM	FISH
miR-30a	CY3-TGTAAACATCCTCGACTGGAAG-CY3	FISH
Bio-NC	Biotin-AAACCCCACTAAAACACTCACCAAGACCTCAACCCAAAAAGGCATAATTAA	RNA pull-down
Bio-circMTND5	Biotin-AAATTAATTATGCCTTTTTGGGTTGAGGTCTTGGTGAGTGTTTTAGTGGGG	RNA pull-down

FISH, Fluorescence in situ hybridization; circ-NC, negative control of circMTND5 on FISH; MIR-NC, negative control of MIR6812 on FISH; Bio-NC, biotinylated negative control.

Supplemental Table 5. Antibodies used in immunostaining and Western blotting

Antibodies	Company	Catalog	Host	Dilution
UCP2	Abcam	Ab97931	Rabbit	1:200 (IF) and 1:1000 (WB)
PGC-1α	Abcam	Ab54481	Rabbit	1:300 (IF) and 1:1000 (WB)
OPA1	Abcam	Ab157457	Rabbit	1:200 (IF)
COL3	Acris	AF5810	Mouse	10ug/ml (IF) and 1:1000 (WB)
FN	Santa Cruz	sc18825	Mouse	10ug/ml (IF) and 1:1000 (WB)
COX IV	Servicebio	GB11250	Rabbit	1:500 (IF)
AGO2	Zenbio	381637	Rabbit	1:500 (IF)
α-TUBULIN	CST	2148	Rabbit	1:1000 (WB)
Alexa Fluor® 488	Thero Fisher Scientific	A21202/A21203	Donkey	1:300
Alexa Fluor® 594	Thero Fisher Scientific	A21207/A20206	Donkey	1:300

UCP2, uncoupling protein 2; PGC-1α, peroxisome proliferator-activated receptor gamma coactivator 1-alpha; COL3, collagen type III; FN, fibronectin; COX IV, cytochrome C oxidase subunit IV; OPA1, optic atrophy 1; AGO2, Argonaute 2; IF, immunofluorescent staining; WB, Western blotting.

Supplemental Table 6. 32 significantly decreased circRNAs

CircRNAID	P-value	Fold Change	Source	GeneName	Predicted sequence length
chrM:14068-14413+	1.91E-05	70.70488	novel	MTND5	346
chrM:14131-15754-	0.00036	39.82782	novel	JA760602	1624
chrM:14131-15754+	0.006914	23.96471	novel	MTND5	1624
chrM:15492-15740-	0.000241	23.77568	novel	JA760602	249
chrM:14068-14923+	0.006209	19.31961	novel	MTND5	856
chrM:14068-14446+	0.00085	19.16315	novel	MTND5	379
chrM:14056-14263-	0.005933	18.81315	novel	JA760602	208
chrM:14068-14923-	0.048645	17.05835	novel	JA760602	856
chrM:13856-14421-	0.005469	14.36966	novel	JA760602	566
chrM:14056-14377-	0.01469	10.83938	novel	JA760602	322
chrM:14074-14394-	0.006651	10.18194	novel	JA760602	321
chrM:9044-11249-	0.009304	9.840272	novel	JA760602	2206
chrM:5169-6247-	0.014753	8.885925	novel	JA429830	1079
chrM:13847-14413-	0.03345	8.625013	novel	JA760602	567
chrM:14068-15403-	0.006879	8.224576	novel	JA760602	1336
chrM:7749-8685-	0.037152	7.79038	novel	JA760602	937
chrM:14056-14263+	0.038915	7.749908	novel	MTND5	208
chrM:4198-6296-	0.032663	6.958123	novel	JA429830	2099
chrM:7749-8685+	0.037808	6.936876	novel	OK/SW-cl.16	937
chrM:14213-15778+	0.040172	6.503446	novel	cytochrome b	1566
chrM:5483-6492-	0.039652	5.793912	novel	JA429830	1010
chrM:13940-15335-	0.042623	5.740001	novel	JA760602	1396
chrM:2227-8441-	0.031038	4.809761	novel	JA429830	6215
chrM:3323-3598-	0.031496	4.809761	novel	TVAS5	276
chrM:5169-8175-	0.03985	4.36092	novel	JA429830	3007
chrM:5169-8175+	0.041941	4.201714	novel	OK/SW-cl.16	3007
chrM:5310-8625-	0.041101	4.005007	novel	JA429830	3316
chr15:64791492-64792365-	0.046044	3.743301	novel	ZNF609	874
chr7:142105140-142162950-	0.038978	3.743301	novel	CDR3	57811
chrM:3526-5337-	0.042366	3.568056	novel	MT-TQ	1812
chrM:14005-14277-	0.049895	3.334902	novel	JA760602	273
chrM:14330-14923+	0.045173	3.334902	novel	cytochrome b	594
